# The current status of pharmaceutical care provision in tertiary hospitals: results of a cross-sectional survey in China

**DOI:** 10.1186/s12913-020-05371-7

**Published:** 2020-06-08

**Authors:** Xiaobo Guo, Dongning Yao, Jie Liu, Yuankai Huang, Yitao Wang, Wenbing Yao

**Affiliations:** 1 National Development Research Center of Licensed Pharmacist, China Pharmaceutical University, Nanjing, China; 2 Institute of Chinese Medical Sciences, University of Macau, Macao SAR, Macao China

**Keywords:** Pharmaceutical care, Tertiary hospitals, China, Current status, Cross-sectional survey

## Abstract

**Background:**

Pharmaceutical care is attached with increasing importance around the world due to its clinical and economical effects. Tertiary hospitals are equipped with the richest healthcare resources and pioneer in the implementation of pharmaceutical care. Understanding current status of pharmaceutical care provision in tertiary hospitals not only helps to improve the practice in tertiary hospitals but also guide the development of pharmaceutical care in secondary and primary health institutions.

**Method:**

Data of a cross-sectional survey were used. The cross-sectional survey was conducted from July 2015 to June 2016, involving 520 hospital directors, 740 clinical pharmacists, 1298 physicians, 778 dispensing pharmacists and 3096 patients from 292 hospitals of 23 provinces, 4 municipalities in mainland China. The survey aimed to comprehensively investigate the current status of pharmaceutical care and health care professional’s understanding of clinical pharmacist system in tertiary hospitals. This study reports results pertaining to current status of pharmaceutical care, including pharmacy department practice rules, guidelines and records, application of rational drug use software, staffing and working arrangement of clinical pharmacists and physicians, patients’ satisfaction toward pharmaceutical care.

**Results:**

A majority of the tertiary hospitals established clinical pharmacist system (84.2%), clinical pharmacist management rules (89%), clinical pharmacists’ working ethics (89%) and applied clinical rational drug use software (93.8%). However, a number of hospitals did not establish a performance evaluation system (37%) and payment rules for pharmaceutical care (81.9%). Most of the clinical pharmacists met the educational background set by the government. Averagely there were 8.3 clinical pharmacists per hospital and 90.7% of the tertiary hospitals had at least five full-time clinical pharmacists. Pharmaceutical care services provided include checking prescriptions, making treatment plans and joining clinical rounds and etc. Both physicians and patients were generally satisfied with pharmaceutical care services provided.

**Conclusion:**

China has made progress in pharmaceutical care provision, but problems such as lack of rules for pharmaceutical care payment and a performance evaluation system, a monotonous variety of pharmaceutical care activities remain unsolved. Policy makers and hospitals directors are suggested to pay more attention to these problems.

## Background

Pharmaceutical care is a term frequently used in health literature and the activity of patient care. More than one definition of pharmaceutical care has been put up and the most widely accepted definition of pharmaceutical care is “Pharmaceutical care is the responsible provision of drug therapy for the purpose of achieving definite outcomes which improve a patient’s Quality of Life” put up by Hepler and Strand in 1990 [1]. A more modern definition was also given by the Pharmaceutical Care Network Europe in 2013, which is “Pharmaceutical care is the pharmacist’s contribution to the care of individuals in order to optimize medicines use and improve health outcomes” [2]. As there are different activities and services provided in the process of pharmaceutical care, no specific activities are mentioned as part of definitions [2].

Over the years, pharmaceutical care has been attached with increasing importance for improving clinical outcomes and lightening economic burden [3] and is provided in the US, the UK, Canada, and many other countries around the world [4, 5]. The term “pharmaceutical care” was introduced to China in the early 1990s. Its implementation, however, did not start until the late 1990s [6]. The most common pharmaceutical care activities are working as a member of the health-care team, checking prescriptions or participating in routine clinical rounds and etc. [7]

Unlike western countries where pharmaceutical care is mainly provided in community pharmacies [1], in China it is mainly provided in secondary and tertiary hospitals. Secondary hospitals, located at counties or districts, mainly provide medical services to local residents. Tertiary hospitals are the highest level of hospital in China and include national, provincial, municipal and medical-school-affiliated hospitals. Compared with secondary hospitals, tertiary hospitals are equipped with the richest healthcare resources [8] and pioneer in the implementation of pharmaceutical care [9]. The practice of pharmaceutical care in tertiary hospitals are supposed to be the best among all hierarchy of hospitals. Therefore, understanding current status of pharmaceutical care provision in tertiary hospitals not only helps to improve the practice in tertiary hospitals but also guide the development of pharmaceutical care in secondary and primary health care institutions.

Literature retrieval of pharmaceutical care provision or clinical pharmacy services provision indicate a lack of large-scale studies focusing on the situation in tertiary hospitals. Hu (2008) conducted his series of researches and investigated medical staff’s attitudes toward and practices of pharmaceutical care [9, 10]. Yao et al. (2017) conducted a national survey to investigate the current status of pharmaceutical care in county hospitals (secondary hospitals) in China [11]. These studies did not specifically investigate the state of pharmaceutical care provision in tertiary hospitals. Xing and Li’s study (2016) targeted at the status of pharmaceutical care in tertiary hospitals [12], but the sample size is too small to reflect the whole picture. Therefore, a cross-section study of large sample targeting tertiary hospitals is needed.

## Method

### Design and setting of the study

Data of a cross-sectional survey were used. The survey was conducted from July 2015 to June 2016, involving 520 hospital directors, 740 clinical pharmacists, 1298 physicians, 778 dispensing pharmacists and 3096 hospitalized patients from 292 hospitals of 23 provinces, 4 municipalities in mainland China. The survey was conducted to comprehensively investigate the current status of pharmaceutical care and health care professional’s understanding of clinical pharmacist system in tertiary hospitals in China. This article only reports results about current status of pharmaceutical care.

### Questionnaire development

The design of questionnaires was based on results of literature research and expert consultation. To begin with, literature review was performed to collect the standards, requirements, norms, guidelines and the current status of pharmaceutical care provision. The collected information include highly referred literature in the fields [1, 13], guidance documents issued by the American Society of Health-system Pharmacists (ASHP) and the American College of Clinical Pharmacy (ACCP) [14–20] and the latest laws and regulations in China, such as the *Good Pharmacy Practice* and the *Rules of Pharmaceutical Affairs Management of Medical Institutions (Rules of Pharmaceutical Affairs)*. Experts in related fields were also interviewed to collect information about the current situation and the latest developments of pharmaceutical care in China.

Based on the result of literature review and expert interviews, a set of initial questionnaires was formed. There were all together six questionnaires targeted at different participant groups: Q1 was designed to collect the basic information of hospitals, including the pharmacy department practice rules, guidelines and records, the application of rational drug use software, the application of management rules, the staffing of medical staff and other information pertaining to pharmaceutical care [11, 21]. Q1 would be answered by administrators of pharmacy department (see Additional file 1 for questionnaire 1).

Q2, Q3, Q4, Q5, Q6 were designed to investigate the current status of pharmaceutical care provision from the perspective of clinical pharmacists, physicians, hospital directors, patients, and pharmacists who did not serve as clinical pharmacist, mainly dispensing pharmacist (see the Additional file 1 for questionnaire 2 ~ 6). These groups of people were chosen because they were directly or indirectly involved in pharmaceutical care and knew the situation. Each questionnaire contains two parts. Part one is demographic part including gender, age, educational degree, professional title, working experience and etc. Part two include understanding of pharmaceutical care, attitudes toward clinical pharmacist system, working arrangements and actual practice of pharmaceutical care and etc.

The six interrelated questionnaires were designed to comprehensively investigate the status quo of pharmaceutical care and participants’ attitudes and opinions toward pharmaceutical care. This article only reports results pertaining to actual practice of pharmaceutical care and other results will be captured by future articles. Information used in this study were mainly from questionnaires answered by administrators of pharmacy department and clinical pharmacists. A few questions from patients and physicians were also used, while the survey result of hospital directors and dispensing pharmacists were not used because of less relevance.

A pilot survey was conducted from March to April 2015 at five tertiary hospitals in Nanjing, Jiangsu Province, China. Reliability of the questionnaires were tested by α-coefficient method and validity of the questionnaire was tested by KMO test and Bartley sphere test. The α reliability coefficient of each questionnaire was greater than 0.8, the KMO test coefficients was greater than 0.5, and the *P* values was less than 0.05, indicating reliability and validity of questionnaires. Minor language adjustment was made when respondents indicated challenge of comprehension.

### Sampling

There are 23 provinces, 4 municipalities and 5 autonomous regions in mainland China. To ensure sample representativeness and minimize potential bias, a four-stage sampling strategy was adopted. The details were as follows:

① The first stage: 23 provinces and 4 municipalities were included as the primary sample units and 5 autonomous regions (Xinjiang Uygur Autonomous Region, Inner Mongolia Autonomous Region, Guangxi Zhuang Autonomous Region, Ningxia Hui Autonomous Region, Tibet Autonomous Region) were excluded from the sample due to difficulty in data collection.
② The second stage: provinces have jurisdiction over both municipal-level and prefecture-level cities. This study divided all municipal-level cities into three groups according to the per capita disposable income level; Selected one prefecture-level city out from each group by random number method, and included a total of 81 prefecture-level cities plus 4 municipalities as the second stage sample units;
③ The third stage: At least one tertiary hospital was selected in each of the 81 prefecture-level cities and at least two tertiary hospitals were selected in each municipality;
④ The fourth stage: In each enrolled tertiary hospital, at least one hospital director, one administrator of pharmacy department, three clinical pharmacists, three physicians, three dispensing pharmacists and five patients were selected.

### Data collection, entry and analysis

A total of 62 undergraduate students majoring in clinical pharmacy were recruited as data collectors. All of them were trained at the same time by the researcher in the form of lecture and Q&A to grasp the background, purposes and methods of the survey. The survey was conducted from July 2015 to June 2016. Two data collectors were dispatched to each province or municipality. First, the data collectors visited directors of each hospital, made self-introduction, invited the directors to participate the survey and asked permission to approach other health professionals and patients in this hospital. If the directors agreed, he/she would first sign a consent form and then be provided with an electronic devise installed with “Interview Master”, a survey app devised for this survey and given brief instructions about how to complete the questionnaire on the app.

After the directors finished the questionnaire, data collectors were introduced to other potential participants. Participants were approached at their working place or other places for their convenience. Administrators of pharmacy department were often visited in their offices; Clinical pharmacists were visited in the pharmacy or inpatients building; Dispensing pharmacists were visited in the pharmacy; Physicians were visited in the consulting room and patients in the inpatient building. And patients in the inpatients building were randomly invited to join the survey. Purpose and contents of the survey would be informed to potential participants and consent form would be signed before survey. All the survey would be conducted on the electronic devise “Interview Master”. After each participant finished the survey, the data were uploaded to the server at China Pharmaceutical University.

After complementing the investigation, the data were entered to EpiData 3.1 and subject to data verification. Descriptive analyses were reported. Categorical variables were summarized as the number of participants and the corresponding percentage (missing values excluded). Continuous variables were presented as the mean and SD of the number. Data analysis was performed by SPSS 22.0.

## Results

### Demographic characteristics of the participants

Because participants of the survey were approached by our data collectors in a face to face way and only those who agreed to participate the survey would answer the questionnaire, therefore the response rate is 100%. A total of 292 hospitals participated in the survey. According to the *China Health and Family Planning Statistical Yearbook 2016*, there are altogether 2023 tertiary hospitals in China in 2015 [22], thus our sample represent 13.8% of the tertiary hospital. Altogether, 520 questionnaires from hospital directors, 740 questionnaires from clinical pharmacists, 1298 questionnaires from physicians, 778 questionnaires from dispensing pharmacists and 3096 questionnaires from patients were collected.

74.6% of the clinical pharmacists were female and 25.4% of them were male. Most have a postgraduate degree (54.6%) or bachelor’s degree (36.9%). Their job rank (junior versus intermediate level, vice-senior, senior level) were mainly junior level (30.7%) or intermediate level (47.2%). Most of the clinical pharmacists had a working experience (60.8%) of 1 ~ 5 years. 38.2% of physicians were males and 61.8% of them were females. 94.8% of the physicians had a bachelors’ degree or above. 79.1% of the physicians had a working experience of more than 5 years. 67.7% of hospital directors were male and 32.2% were female. Most of them (83.1%) were elder than 40 years old. 92.2% of them had a bachelor’s degree or above. For dispensing pharmacists, 29.9% were male and 70.1% were female. Most of them (37.8%) aged 20 ~ 29. 85.6% of them has a bachelors’ degree or above. 65.9% of them has a working experience of longer than 5 years. As for patients, 46.1% were male and 53.9% were female. Most of them (42.3%) were elder than 50. Only 22.1% of the patients had a bachelor’s degree or above (Table 1).

**Table 1:** Demographic Characteristics of the Participants

	Clinical pharmacists (***n*** = 740)		Physicians (***n*** = 1298)		Hospital directors (520)		Dispensing pharmacists (***n*** = 778)		Patients (***n*** = 3096)	
Gender										
Male	188	25.4%	496	38.2%	352	67.7%	233	29.9%	1427	46.1%
Female	552	74.6%	802	61.8%	168	32.3%	545	70.1%	1669	53.9%
Age group										
< 20	/	/	/	/	/	/	/	/	113	3.6%
20–29	274	37%	280	21.6%	22	4.2%	294	37.8%	614	19.8%
30–39	291	39.3%	484	37.3%	44	8.5%	225	28.9%	524	16.9%
40–49	107	14.5%	365	28.1%	200	38.5%	199	25.6%	534	17.2%
≥ 50	68	9.2%	169	13%	254	48.8%	60	7.7%	1309	42.3%
Missing value	0	0%	0	0%	0	0%	0	0%	2	0.1%
Educational level										
Below bachelor’s degree	28	3.8%	68	5.2%	46	8.8%	113	14.5%	/	88%
Bachelor’s degree	273	36.9%	576	44.4%	210	40.4%	468	60.2%	/	22.1%(BA^a^ and above)
Master’s degree	404	54.6%	432	33.3%	124	23.8%	167	21.5%	/	/
Doctor’s degree	35	4.7%	222	17.1%	140	26.9%	30	3.9%	/	/
Job rank										
Primary level	227	30.7%	/	/	0	0%	391	50.3%	/	/
Intermediate level	349	47.2%	/	/	28	5.4%	216	27.8%	/	/
Vice-senior level	102	13.8%	/	/	87	16.7%	147	18.9%	/	/
Senior level	62	8.4%	/	/	405	77.9%	24	3.1%	/	/
Years of working										
1–5 years	450	60.8%	268	20.6%	268	20.6%	265	34.1%	/	/
6–10 years	147	19.90%	218	16.80%	218	16.8%	11	20.7%	/	/
11–19 years	20	2.70%	290	22.40%	290	22.4%	161	20.7%	/	/
More than 20 years	120	16.20%	521	40.20%	521	40.2%	191	24.6%	/	/
Missing value	0	0	1	0.10%	1	0.1%	0	0%	/	/

^a^*BA* Bachelor’s degree

### Pharmacy department practice rules, guidelines and records

In China, hospitals’ pharmacy department is required to establish and apply rules, guidelines, and records of pharmaceutical care [11]. Guidelines for implementing pharmaceutical care activities, or other management rules such as formal evaluation of clinical pharmacists helps to define the role of clinical pharmacists and provide evidence-based support on how to implement pharmaceutical care activities [7].

Table 2 shows the survey results of pharmacy department written goals and objectives or written rules for implementing pharmaceutical care. Overall, a majority of the hospitals established a clinical pharmacist system (*n* = 246, 84.2%), clinical pharmacist management rules (*n* = 260, 89%) and clinical pharmacists’ working ethics (*n* = 260, 89%), indicating that an intention to promote the development of pharmaceutical care and efforts made to a construct rules and systems. However, there were still a number of hospitals (*n* = 108, 37%) did not established a clinical pharmacists’ performance evaluation system. In terms of payment for pharmaceutical care, the survey result shows that only 18.1% of the hospitals (*n* = 134) charge fees for pharmaceutical care services. The services charged include therapeutic drug monitoring (*n* = 161, 21.8%) and consultation (*n* = 208, 28.1%) etc.

**Table 2:** System Construction and Hardware Equipment

	Frequency	Percentage
Have the hospital established a clinical pharmacist system?		
No	46	15.8%
Yes	246	84.2%
Have the hospital established clinical pharmacist management rules?		
No	16	5.5%
Yes	260	89%
Absent	16	5.5%
Have the hospital established clinical pharmacists’ working ethics?		
No	16	5.5%
Yes	260	89%
Absent	16	5.5%
Have the hospital established a clinical pharmacist performance evaluation system?		
No	108	37%
Yes	160	54.8%
Absent	24	8.2%
Is Are there any services provided by clinical pharmacists charged for fee?		
No	606	81.9%
Yes	134	18.1%
Services charged for fees include		
Reviewing physicians’ orders	21	2.8%
Pharmaceutical monitoring	84	11.4%
Patient medication guidance	38	5.1%
Therapeutic drug monitoring	161	21.8%
Pharmacy rounds	64	8.6%
Consultation	208	28.1%
Gene detection	2	0.3%
Drug genetics test	4	0.5%
Drug counseling	4	0.5%

### The application of rational drug use software

The application of rational drug use software is also believed to affect the efficacy of pharmaceutical care. Rational drug use software, also known as the sound drug formulary system, is an administration system of drug information based on clinical medication databases [11]. It functions as a part of hospitals’ information system and provides information on diagnosis and clinical drug use. Rational drug use software has become an essential tool for modern clinical pharmacy service [23]. According to the survey, most hospitals (93.80%) applied clinical rational drug use software.

### The staffing and working arrangement of clinical pharmacists

According to the Rules of Pharmaceutical Affairs, a tertiary hospital should be equipped with at least five full-time clinical pharmacists. Our survey data show that 90.7% of the tertiary hospitals met this requirement. The mean number of clinical pharmacists per hospital was 8.3 with the standard being 3.7. As shown in Table 3, averagely, there were 6.3 pharmacy professionals per 100 beds, and 0.43 clinical pharmacists per 100 beds. The proportion of full-time clinical pharmacists to all clinical pharmacists in the hospital was 8.3%, indicating that clinical pharmacists accounted for a small proportion of the pharmaceutical professionals.

**Table 3:** The Staffing of Pharmacy Professionals

	Mean	Standard deviation
Number of clinical pharmacists	8.3	3.7
Number of the pharmaceutical professionals in the hospital	105.5	44.3
The proportion of hospital pharmacy professionals in the hospital’s health professionals	6.3%	7.4%
The proportion of full-time clinical pharmacists to all clinical pharmacists in the hospital	8.3%	3.7%
Percentage of clinical pharmacists per 100 beds	0.4%	0.2%

As shown in Table 4, the working time of clinical pharmacists in the ward was mainly within 2–4 h/day (32.8%) or 4–6 h/day (35.8%). 39.3% of the clinical pharmacists spent more than 80% of working time on clinical work and quite a lot of clinical pharmacists spent 40–60% of the time on clinical work (28.5%). The main duty of their clinical work was checking prescriptions (25.9%), making treatment plans (17.3%) and joining clinical rounds (15.7%) and etc.

**Table 4:** Working arrangement of clinical pharmacists

	Frequency	Percentage
The time clinical pharmacist spent in ward		
More than 6 h/day	125	16.9%
4–6 h/day	265	35.8%
2–4 h/day	243	32.8%
Less than 2 h/day	107	14.5%
The proportion of the time clinical pharmacy-related work occupied to all the working hours		
Less than 20%	55	7.4%
20–40%	46	6.2%
40–60%	211	28.5%
60–80%	137	18.5%
More than 80%	291	39.3%
Focus of clinical work		
Dispensing	46	6.2%
Drug supply	42	5.7%
Pharmacy management	8	1.1%
Pharmaceutical research	16	2.2%
Training and teaching	28	3.8%
Checking prescriptions	192	25.9%
Drug monitoring	62	8.4%
Making treatment plans	128	17.3%
Pharmacy consultation	16	2.2%
Joining clinical rounds	116	15.7%
Medical consultation	28	3.8%
Medication guidance	46	6.2%
Pharmaceutical monitoring	60	8.1%
Others	12	1.6%

### Physicians and patients’ satisfaction toward pharmaceutical care

As shown in Table 5, 49.2% of the physicians believed that clinical pharmacists were very helpful to their work, and 47.7% think it was quite helpful. 37.1% of the physicians totally adopted suggestions on medication or dosing regimens given by clinical pharmacists, while more than half of the physicians (60.5%) indicated that they partially accept suggestions given by clinical pharmacists.

**Table 5:** Outcomes of pharmaceutical care

	Frequency	Percentage
(Physician) Do you think clinical pharmacists are helpful to your work?		
Very helpful	639	49.2%
Quite helpful	619	47.7%
Not sure	30	2.3%
Not helpful	6	0.5%
Be more of a hindrance than a help	4	0.3%
(Physician) What is your attitude towards clinical pharmacists’ medication suggestions?		
Totally adopt	481	37.1%
Partially adopt	785	60.5%
Neutral	23	1.8%
Rarely adopt	9	0.7%
Absolutely do not adopt	0	0%
(Patients) Pharmaceutical care received		
Disease history and medication history	2330	75.3%
Drug guidance	2416	78%
Medication consultation	2360	76.2%
Rounds	1962	73.5%
(Patients) Satisfaction toward pharmaceutical care		
Disease history and medication history examination	1813	77.8%
Drug guidance	1965	81.3%
Medical consultation	1868	79.2%
Rounds	1494	76.1%
(Patients) Dissatisfaction with pharmaceutical care		
Disease history and medication history	44	1.9%
Drug guidance	56	2.3%
Medical consultation	91	3.9%
Rounds	65	3.3%

Most patients had the experience of receiving pharmacy care services, and were satisfied with it. Only a small number of patients were dissatisfied with pharmaceutical care services. Among all the pharmaceutical care services provide, medication guidance wins satisfaction of most people (81.3%).

## Discussion

This study involves participants from 23 municipalities of China and therefore is of good generalizability. It is found through the survey that a majority of the tertiary hospitals have established clinical pharmacist system (84.2%), clinical pharmacist management rules (89%) and clinical pharmacists’ working ethics (89%). However, a number of hospitals have not established a performance evaluation system (37%) and payment rules for pharmaceutical care (81.9%). This result corresponds with a study conducted in Shenyang province of China, which find that that 64.4% of the hospitals does not establish a performance evaluation system for clinical pharmacists and 53.1% does not establish pharmaceutical care management rules [24]. Reasons for lacking these management rules might be that it has not been long since the establishment of clinical pharmacist system, and there is still a lack of consensus on these rules [25]. Take payment rules for pharmaceutical care as an example, despite the evidence that pharmaceutical care improves patient outcomes and provide solid return on investment [26], pharmaceutical care is still provided for free in many hospitals [27], where the practice of pharmaceutical care is regarded as more of an expenditure than profit, and lacks sustainability [7]. China is now working on setting the payment standards for pharmaceutical care and this policy have been carried out as pilot in a few areas [28]. Future efforts should be made to expand payment for pharmaceutical care and change the perception that “pharmaceutical care is free” [29].

Apart from pharmacy department practice rules, clinical pharmacists themselves, as the main provider of pharmaceutical care, are also important for pharmaceutical care provision. According to our survey, 91.5% of the pharmacists have received a bachelor’s degree or above, which is minimum education requirement set by the government. Compared with Shen and Wang’s finding that 68.5% of pharmacists have received a bachelor’s degree or above [30], our research find an impressive increase of 23%, indicating an improvement in pharmacy staff’s educational background. This might be explained by the Chinese governments’ emphasis on the importance of pharmacy education and the hospitals’ strict control of educational background when hiring new pharmacy personnel. In China, the Ministry of Health is working to establish standardized entry level criteria for clinical pharmacists via the Licensed Pharmacist Law, and it is expected that only graduate students with at least a bachelor’s degree in clinical pharmacy or pharmacy will able to be qualified as clinical pharmacists by passing a national examination [31]. This is different from many countries where PharmD is required.

Besides, it is an interesting phenomenon that the duty of clinical pharmacists mainly focuses on the simplest form of pharmaceutical care activities, like checking prescriptions, making treatment plans and joining clinical rounds, while other activities, such as medical consultation, drug monitoring were less frequently conducted. This indicates the need to expand the activities of pharmaceutical care activities and provide more forms of pharmaceutical care activities.

As for satisfaction toward pharmaceutical care, 96.9% of the physicians agreed that the work of clinical pharmacists is very\quite helpful. Most of the patients are satisfactory with pharmaceutical care, which fits Zhang’s finding that patients are generally satisfactory with pharmaceutical care [32].

This study has limitations. First, our study focus on pharmaceutical care provision of tertiary hospitals and therefore may not be external to secondary and primary health care institutions. Second, participants of the survey were approached by data collectors in a face to face way. Only those agreed to participate in the survey would answer the questionnaire and therefore the response rate is 100%. This may to some degree cause risk of bias and lead to overestimation because participants who are more confident in their practice of pharmaceutical care may be more willing to cooperate. Third, some issues derived from the discussion require additional data for further discussion. Take reasons for lacking of well-established management rules as an example, all assumptions provided in the discussion are merely based on the limited data of our survey, our future researches will go further and give evidence-based explanation to issues derived from this study.

## Conclusion

This study is a national survey involving 292 tertiary hospitals with the aim to investigate the current status of pharmaceutical care in China. Through the survey, it is found that most tertiary hospitals in China are attaching increasing importance to pharmaceutical care and are equipped with basic software and hardware facilities facilitating the provision of pharmaceutical care. However, problems still exist. For example, the lack of rules for pharmaceutical care payment, the lack of a performance evaluation system and a monotonous variety of pharmaceutical care activities are still waiting to be solved. Policy makers and hospital directors should to pay attention to these problems.

## Supplementary information

**Additional file 1.**

## Data Availability

The datasets used and analyzed during the current study are available from the corresponding author on reasonable request.

## Abbreviations

Q&A: Questions and answers
