# Supplementary material for: The current status of pharmaceutical care provision in tertiary hospitals: results of a cross-sectional survey in China
Source: BMC Health Serv Res. 2020 Jun 8;20:518. doi: 10.1186/s12913-020-05371-7 (PMC7282101; doi:10.1186/s12913-020-05371-7)
Supplement: Supplementary file 1 — Additional file 1. [file 12913_2020_5371_MOESM1_ESM.docx]

Supplementary Appendix

This appendix is about the questionnaires used in the survey. The original questionnaires were in Chinese and were translated into English. The six interrelated questionnaires were designed to comprehensively investigate the status quo of pharmaceutical care and participants’ attitudes and opinions toward pharmaceutical care. This article only reports results pertaining to actual practice of pharmaceutical care and other results will be captured by future articles. We provided the complete questionnaire of the original survey and marked questions relevant to this study in red.

Table of Contents

[Appendix1 Basic Information of Surveyed Hospitals for the Administrator of Pharmacy Department 3](#_Toc35372036)

[Appendix2 A Survey on the Current Status of Pharmaceutical Care Provision in Tertiary Hospitals of China for Clinical Pharmacists 8](#_Toc35372037)

[Appendix3 A Survey on the Current Status of Pharmaceutical Care Provision in Tertiary Hospitals of China for Physicians 15](#_Toc35372038)

[Appendix4 A Survey on the Current Status of Pharmaceutical Care Provision in Tertiary Hospitals of China for Hospital Directors 20](#_Toc35372039)

[Appendix5 A Survey on the Current Status of Pharmaceutical Care Provision in Tertiary Hospitals of China for Inpatients 25](#_Toc35372040)

[Appendix6 A Survey on the Current Status of Pharmaceutical Care Provision in Tertiary Hospitals of China for Dispensing Pharmacist 29](#_Toc35372041)

# Questionnaire 1 Basic Information of Surveyed Hospitals for Administrators of Pharmacy Department

**A Survey on the Current Status of Pharmaceutical Care Provision**

**in Tertiary Hospitals of China**

**Basic Information of Surveyed Hospitals**

**(For Administrators of Pharmacy Department)**

**Basic information**

1. Name: Phone number:
2. Name of the hospital you work for:
3. Hospital type:A.general tertiary hospital B.specialized tertiary hospital
4. Number of beds in hospital:_______
5. The department of pharmacy is administrated by:
6. Director B. Managing/Vice director C. Secretary D. Deputy secretary

E. Head of medical administration F. Other:

1. Number of technical personnel in pharmacy is , the percentage of which in all technical personnel is %.

6.1 Number of personnel

aged from 20 to 35: ;aged from 36 to 49: ;aged over 50: .

6.2 Number of personnel responsible for

Drug dispensing: ; Drug supply: ;

Pharmacy administration: ;

Specialized clinical pharmaceutical service: ;

Academic research: ; Other aspects: .

6.3 Number of personnel with

Bachelor’s degree: ; Master’s degree: ; Doctor’s degree: .

6.4 Number of personnel ranked at

Junior level: ; Intermediate level: ; Senior level: .

1. Is the hospital one of the NHFPC training bases for clinical pharmacists?

A.Yes B.No

7.1 The year when the hospital was certificated as NHFPC training base for clinical pharmacists:______

7.2 The number of programs that had been established by the 30^th^ June of 2015: , including: .

1. Is the hospital one of NHFPC training bases for clinical pharmacists education?

A.Yes B.No

The year when the hospital was certificated as NHFPC training base for clinical pharmacists education:__________

1. Number of full-time clinical pharmacists: ;

Number of those who obtain NHFPC training certificate for clinical pharmacists: ;

Number of those who obtain NHFPC training certificate for clinical pharmacists education: .

1. The division where full-time clinical pharmacists work and the number of clinical pharmacists in each division:

| Clinical division | Number | Clinical division | Number |
| --- | --- | --- | --- |
| 🞏 Respiratory medicine |  | 🞏 Cardiovascularology |  |
| 🞏 Endocrinology |  | 🞏 Anti-tumor medication |  |
| 🞏 Gastroenterology |  | 🞏 Neurology |  |
| 🞏 Pain-management |  | 🞏 ICU |  |
| 🞏 Anti-infective medication |  | 🞏 Immune system medication |  |
| 🞏 Enteral and parenteral nutrition |  | 🞏 Gynaecology and obstetrics |  |
| 🞏 Anticoagulant medication |  | 🞏 General clinical pharmacists |  |
| 🞏 Other division__________ |  | 🞏 Other division_______ |  |

1. Have the hospital established a clinical pharmacist system?

A.Yes B.No

1. Which of the following departments are responsible for the performance assessment and bonus allocation for clinical pharmacists?

A.Department of pharmacy B.Clinical department C.Both D.Other department

1. Have the hospital applied the Rational drug use software?

A.Yes B.No

If the answer is “A”, then it was manufactured by:

1. Health&Beauty Group inc B.Datong C.Other brand
2. Is there any specialized office for clinical pharmacists in their working region?
3. Yes B.No
4. Have the hospital established clinical pharmacist management rules?

A.Yes B.No

If the answer is “A”, then it was established by:

1. Department of pharmacy
2. Department of medical administration
3. Department of pharmacy and department of medical administration
4. Other department
5. Have the hospital established clinical pharmacists’ working ethics?

A.Yes B.No

If the answer is “A”, then it was established by:

1. Department of pharmacy

B. Department of medical administration

C. Department of pharmacy and department of medical administration

D. Other department

1. Have the hospital established a clinical pharmacist performance evaluation system?

A.Yes B.No

If the answer is “A”, then they were issued by:

1. Department of pharmacy

B. Department of medical administration

C. Department of pharmacy and department of medical administration

D. Other department

**Interviewer’s name:** **Phone number:**

**Time when the form is finished:**

**Time to finish the form:**

**Attachment:Basic Information of Specialized Clinical Pharmacists**

| NO. | Name | Age | Gender | Highest education background | First major | Major at highest education background | Rank | Division | Working Year | Date when certificated as a clinical pharmacist by NHFPC | Date when certificated for clinical pharmacist education by NHFPC | E-mail address |
| --- | --- | --- | --- | --- | --- | --- | --- | --- | --- | --- | --- | --- |
|  |  |  |  |  |  |  |  |  |  |  |  |  |
|  |  |  |  |  |  |  |  |  |  |  |  |  |
|  |  |  |  |  |  |  |  |  |  |  |  |  |
|  |  |  |  |  |  |  |  |  |  |  |  |  |
|  |  |  |  |  |  |  |  |  |  |  |  |  |
|  |  |  |  |  |  |  |  |  |  |  |  |  |
|  |  |  |  |  |  |  |  |  |  |  |  |  |
|  |  |  |  |  |  |  |  |  |  |  |  |  |
|  |  |  |  |  |  |  |  |  |  |  |  |  |
|  |  |  |  |  |  |  |  |  |  |  |  |  |
|  |  |  |  |  |  |  |  |  |  |  |  |  |
|  |  |  |  |  |  |  |  |  |  |  |  |  |
|  |  |  |  |  |  |  |  |  |  |  |  |  |
|  |  |  |  |  |  |  |  |  |  |  |  |  |
|  |  |  |  |  |  |  |  |  |  |  |  |  |
|  |  |  |  |  |  |  |  |  |  |  |  |  |
|  |  |  |  |  |  |  |  |  |  |  |  |  |
|  |  |  |  |  |  |  |  |  |  |  |  |  |
|  |  |  |  |  |  |  |  |  |  |  |  |  |
|  |  |  |  |  |  |  |  |  |  |  |  |  |
|  |  |  |  |  |  |  |  |  |  |  |  |  |
|  |  |  |  |  |  |  |  |  |  |  |  |  |
|  |  |  |  |  |  |  |  |  |  |  |  |  |
|  |  |  |  |  |  |  |  |  |  |  |  |  |
|  |  |  |  |  |  |  |  |  |  |  |  |  |

No:

# Questionnaire 2 A Survey on the Current Status of Pharmaceutical Care Provision in Tertiary Hospitals of China for Clinical Pharmacists

**A Survey on the Current Status of Pharmaceutical Care Provision in Tertiary Hospitals of China**

(For Clinical Pharmacists)

**Part one: Basic information**

1. Name: Phone number:
2. Name of the hospital you work for:
3. Hospital type:A. General tertiary hospital B. Specialized tertiary hospital
4. Gender: A. Male B. Female
5. Age: A. 20-29 B. 30-39 C. 40-49 D. elder than 50
6. Education: A. Below bachelor’s degree B. Bachelor’s degree

C. Doctor’ s degree D. Master’s degree

Specialty for college:­­________ Specialty for doctor’s program:­­________ Specialty for Doctors program:­­________

1. Rank: A. Junior level B. Intermediate level C. Vice-senior level D. Senior level
2. Years of working:A.1-5 years B.6-10 years C.11-19 years D. More than 20 years
3. Qualifications

A. Specialist clinical pharmacist trained at the national level

B. General clinical pharmacist trained at the national level

C. General clinical pharmacist trained at the provincial level

D. With no qualification

1. Current position ；years of working in current position
2. Training information:
3. Certificated as clinical pharmacists by NHFPC, Date:_______; Speciality:
4. Certificated as advanced clinical pharmacists by NHFPC, Date:
5. Certificated for clinical pharmacists education by NHFPC, Date:

D. Trained for clinical pharmacists abroad: Nation ; Endurance:

E. Other training program:

**Part 2: (Have only one choice unless specially notified)**

1. How much do you know about the development trend of clinical pharmacy in foreign countries?

A. very much B. much C. a little D. very little E. none

1. What do you think of the future development of hospital pharmacy?

（You may have less than two choices）

A. Mainly focus on drug supply

B. Mainly focus on drug dispensing

C. Mainly focus on academic research

D. Mainly focus on rational administration of drugs

E. Mainly focus on pharmaceutical care

1. To what degree are the following factors important for the need of clinical pharmacist? Please mark a"√".

|  | Degree of importance | | | | |
| --- | --- | --- | --- | --- | --- |
|  | Very important | Important | Neutral | A little important | Not important at all |
| Policy incentives |  |  |  |  |  |
| Hospitals’ Attention |  |  |  |  |  |
| Professional ability of the pharmacist |  |  |  |  |  |
| Acceptance of medical staff |  |  |  |  |  |
| Acknowledgement of patients |  |  |  |  |  |

1. Are you optimistic about the career development of clinical pharmacists?
2. very optimistic B. optimistic C. neutral

D. not optimistic E. not optimistic at all

1. How much do you know about "clinical pharmacist system"?

A. Very much B. much C. a little D. little E. none

1. Do you think it is necessary for medical institutions to apply "clinical pharmacist system"?
2. very necessary B. necessary, but the timing is not right

C. doesn’t matter D. not necessary

1. How is the "clinical pharmacist system" implemented in your hospital？

A. smooth B. basically smooth C. not sure D. not smooth E. not implemented

If the answer is E, what factors do you think handicap the implementation of clinical pharmacist system？

1. Policy incentives B. Environment in medical institutions

C. Attention paid by hospital leaders D. Professional ability of the pharmacist

1. Do you think laws and regulations are necessary to regulate clinical pharmacy work?
2. very necessary B. necessary C. neutral

D. Unnecessary E. not necessary at all

If the answer is A or B , in what aspect should the law regulate?

(You may have more than one choices)

A. make clear the role of clinical pharmacists by law

B. make clear the rights and obligations of clinical pharmacists

C. certification and employment of clinical pharmacists

D. cultivation and training of clinical pharmacists

E. assessment of clinical pharmacists

F. salaries and benefits of being a clinical pharmacists

G. others

1. Do you think clinical pharmacists are necessary in clinical environment?
2. very necessary B. necessary C.neutral

D. Unnecessary E. not necessary at all

If the answer is A or B, what are the most important duties and obligations of clinical pharmacists from your perspective of view? （You may have more than one choices）

A. drug supply and drug dispensing B. designing medical treatment

C. review physicians’ orders D. pharmacy consultation

E. pharmaceutical care F. monitoring of adverse drug effects

G. academic research H. others

If the answer is A or B, how many beds should be equipped with one clinical pharmacists?

A.10-20 B.20-50 C.50-100 D.100-200 E. more than200

1. Do you think the staffing of clinical pharmacists in your hospital is reasonable?
2. very reasonable B.quite reasonable C.neutral

D.not reasonable E.not reasonable at all

1. Do you think clinical pharmacists are necessary in clinics?

A. Yes B.No C Not sure

If the answer is A, what is the working focus of clinical pharmacists in clinics?

（You may have less than two choices）

1. Reviewing physicians’ orders B. chronic disease management

C. medication guidance D. medication education

1. Do you think clinical pharmacists are necessary in emergency room?

A. yes B. no C. not sure

If the answer is A, what is the working focus of clinical pharmacists in clinics?

（You may have less than two choices）

A.Reviewing physicians’ orders B. chronic disease management

C. medication guidance D. medication education

1. How did the clinical pharmacists work move on in your hospital?

A. smooth B. basically smooth C. not sure D. not smooth E. not implemented

1. Is your work as a clinical pharmacist helpful for clinical medication?
2. very helpful B. a little helpful C.not sure

D. not helpful E. be more of a hindrance than a help

1. For the past 12 month, to what degree are your suggestions about rational administration of drugs accepted by physicians?

A. less than 20% B.20%-40% C.40%-60% D.60%-80% E. more than 80%

1. What is the attitude of physicians toward the medication suggestions provided by you？
2. Totally adopt B. Partially adopt C. Neutral

D. Rarely adopt E. Absolutely do not adopt

1. What is the attitude of medical staff in clinics toward the medication suggestions provided by you？

A. totally agree B. quite agree C. neutral D. quite disagree E. totally disagree

1. What is the attitude of patients who received service from you for the first time toward your work？

A. totally agree B. quite agree C. neutral D. quite disagree E. totally disagree 30、What is the attitude of patients who have received service from you toward your work？

A. totally agree B. quite agree C. neutral D. quite disagree E. totally disagree

31、In your opinion, what education background is preferred for a clinical pharmacist?

A. pharmacy B.medication C.nursing D. any speciality related to medication

32、In your opinion, what should be the least requirement of education background for clinical pharmacists?

A. Doctor’s degree B. Master’s degree C. Bachelor’s degree D. Others

33、In your opinion, is the one-year-training designed by National Health and Family Planning Commission (now changed into National Health Commission) enough to train a clinical pharmacist as a competent medical staff which suits the clinical needs?

A. enough B. basically enough C. not enough D. not sure

34、Do you think you are qualified as a clinical pharmacists?

A. qualified B. basically qualified C. not sure

D. only if after received training E. not qualified

35、Do you think you have received fair treatment in your position?

A. Yes, I have received fair treatment B. No, I received more than what I deserve

C. No, I deserve more than what I received D. not sure

36、Are there any services in your work charged for fee?

A.Yes B.No

If the answer is A, what are these services?

1. Reviewing physicians' orders B. Pharmaceutical care

C. Patient medication guidance D. Therapeutic drug monitoring

E. Pharmacy rounds F. Consultation G. Other:

37、In your opinion, what services in your work should be charged for fee?

A. Reviewing physicians' orders B. Pharmaceutical monitoring

C. Patient medication guidance D. Therapeutic drug monitoring

E. Pharmacy rounds F. Consultation G.Other:

38、How long do you spend in ward everyday?

A. more than 6 hours per day B.4-6 hours per day

C.2-4 hours per day D. less than 2 hours per day

1. What is the proportion of the time spent in clinical pharmacy-related work occupied in all the working hours?

A. Less than 20% B.20-40% C.40-60% D.60-80% E. More than 80%

40、Focus of your work:__________; the proportion to all your pharmacy related work is:__________.

**Part 3 Clinical Pharmacists’ Perception of Clinical Pharmacy and Assessment of Their Skills and Daily Work in Current Stage**

1: totally disagree 2: basically disagree 3: not sure

4: basically agree 5: totally agree

| **Skills and daily work** | **Degree** | | | | | |
| --- | --- | --- | --- | --- | --- | --- |
| 1.Discover, solve and prevent the potential or practical problems in medication regularly | 1 | | 2 | 3 | 4 | 5 |
| 2.Give suggestions for medication by using the knowledge and skills in pharmacy | 1 | | 2 | 3 | 4 | 5 |
| 3.Provide information consultation about medicine | 1 | | 2 | 3 | 4 | 5 |
| 4.Get new information about medicine and gain new knowledge of medication by literature searching | 1 | | 2 | 3 | 4 | 5 |
| 5.Write medication history and other medical documents | 1 | | 2 | 3 | 4 | 5 |
| 6.Read reports on medical laboratory science, radiography and electrocardiograms, and collect information about the clinical application of medicine accurately | 1 | | 2 | 3 | 4 | 5 |
| 7.Have a good command of the design and evaluation of common diseases’ medication | 1 | | 2 | 3 | 4 | 5 |
| 8.Know the diagnosis and treatment of common diseases | 1 | | 2 | 3 | 4 | 5 |
| 9.Have a deep communication with other medical staff and patients regularly | 1 | | 2 | 3 | 4 | 5 |
| 10.Talk to different people in the right manner and always get desirable information | 1 | | 2 | 3 | 4 | 5 |
| 11.Have a strong sense of responsibility of medication’s safety for patients | 1 | | 2 | 3 | 4 | 5 |
| 12.Follow others’ suggestions | 1 | | 2 | 3 | 4 | 5 |
| 13.Learn from clinical work and make an improvement regularly | 1 | | 2 | 3 | 4 | 5 |
| 14.Handle the emergency in clinical work flexibly | 1 | | 2 | 3 | 4 | 5 |
| 15.Be reputed as an experienced and excellent pharmacist | 1 | | 2 | 3 | 4 | 5 |
| 16.Once be awarded as Working Model, Advanced Individual and Outstanding Pharmacist | 1 | | 2 | 3 | 4 | 5 |
| 17.Professional suggestions could be accepted by medical staff and accordingly change their actions | 1 | | 2 | 3 | 4 | 5 |
| 18.Strive for excellence to become the champion in the industry | | 1 | 2 | 3 | 4 | 5 |
| 19.Discover the irrationality in prescription and could tell whether the medication is complete and accurate regularly | | 1 | 2 | 3 | 4 | 5 |
| 20.Know patients’ ideas, concerns and expectation for medication | | 1 | 2 | 3 | 4 | 5 |
| 21.Not stick to the existing working patterns and make an improvement by questioning and critical thinking | | 1 | 2 | 3 | 4 | 5 |
| 22.Make medication decision together with physicians, patients and their family regularly | | 1 | 2 | 3 | 4 | 5 |
| 23.Undertake the work beyond the duty and improve the work | | 1 | 2 | 3 | 4 | 5 |
| 24.Evaluate the prescription and drug utilization regularly | | 1 | 2 | 3 | 4 | 5 |
| 25.Support the decision-making of clinical medication according to evidence-based pharmacy | | 1 | 2 | 3 | 4 | 5 |
| 26.Convey patients’ condition and medication suggestions to their family timely and accurately | | 1 | 2 | 3 | 4 | 5 |
| 27.Instruct pharmacists or interns on study and give detailed guidance and demonstration regularly | | 1 | 2 | 3 | 4 | 5 |
| 28.Participate in clinical consultation and conference, and provide professional suggestions | | 1 | 2 | 3 | 4 | 5 |
| 29.Prevent the abuse of medicine regularly | | 1 | 2 | 3 | 4 | 5 |
| 30.Promote the rational medication and patient education regularly | | 1 | 2 | 3 | 4 | 5 |
| 31.Analyze and report ADRs according to procedures | | 1 | 2 | 3 | 4 | 5 |
| 32.Choose medication programs based on pharmacoeconomics regularly | | 1 | 2 | 3 | 4 | 5 |
| 33.Participate in bed checks and keep a watch on the change of diseases | | 1 | 2 | 3 | 4 | 5 |
| 34.Provide service of pharmacy informatics | | 1 | 2 | 3 | 4 | 5 |
| 35.Carry out researches on the problems in clinical medication | | 1 | 2 | 3 | 4 | 5 |

**Interviewer’s name:** **Phone number:**

**Time when the form is finished:**

**Time to finish the form:**

# Questionnaire 3 A Survey on the Current Status of Pharmaceutical Care Provision in Tertiary Hospitals of China for Physicians

NO:

**A Survey on the Current Status of Pharmaceutical Care Provision in Tertiary Hospitals of China**

(For Physicians)

**Part one:Basic information**

1. Name of the hospital you work for:
2. Hospital type:A. General tertiary hospital B. Specialized tertiary hospital
3. Name: Phone number:
4. Gender: A. Male B. Female
5. Age: A. 20-29 B. 30-39 C. 40-49 D. elder than 50
6. Education: A. Below bachelor’s degree B. Bachelor’s degree

C. Master’s degree D. Doctor’ s degree

1. Position: A. Resident doctor B. Attending doctor C. Associate professor
2. Professor E. Nurse F. Nurse practitioner

G. Nurse-in-charge H. Associate chief of nursing I. Head nurse

1. Rank: A. Professor B.Associate professor C. Lecturer D Assistant E. None
2. Administrative position: A.department director B. department vice-director

C.head nurse D. doctor E.nurse

1. Years of working:A.1-5 years B.6-10 years C.11-19 years D. More than 20 years
2. Department where you work:
3. Number of Beds in your department:

**Part two: Perception and Evaluation of the Implement of Clinical Pharmacy**

(Have only one choice unless specially notified)

1. How much do you know about the duty of clinical pharmacists?

A.very much B.much C. a little D.have heard but know little E.never heard

If the answer is A, B or C, then you know it from:

1. Clinical pharmacists have been working in your working regions
2. Publication from the department of pharmacy
3. Regulations issued by NHC
4. Publication from medical websites
5. Others

14、What do you think of the future development of hospital pharmacy?

（You may have less than two choices）

A. Mainly focus on drug supply

B. Mainly focus on drug dispensing

C. Mainly focus on academic research

D. Mainly focus on rational administration of drugs

E. Mainly focus on pharmaceutical care

15、Do you think clinical pharmacists are necessary in clinical environment?

1. very necessary B. necessary C.neutral

D. Unnecessary E. not necessary at all

If the answer is A or B, what are the most important duties and obligations of clinical pharmacists from your perspective of view? （You may have more than one choices）

A. drug supply and drug dispensing B. designing medical treatment

C. review physicians’ orders D. pharmacy consultation

E. pharmaceutical care F. monitoring of adverse drug effects

G. academic research H. others

If the answer is A or B, how many beds should be equipped with one clinical pharmacists?

A.10-20 B.20-50 C.50-100 D.100-200 E. more than200

16、To what degree are the following factors important for the need of clinical pharmacist? Please mark a"√".

|  | Degree of importance | | | | |
| --- | --- | --- | --- | --- | --- |
|  | Very important | Important | Neutral | A little important | Not important at all |
| Policy incentives |  |  |  |  |  |
| Hospitals’ Attention |  |  |  |  |  |
| Professional ability of the pharmacist |  |  |  |  |  |
| Acceptance of medical staff |  |  |  |  |  |
| Acknowledgement of patients |  |  |  |  |  |

17、In your opinion, what education background is preferred for a clinical pharmacist?

A. pharmacy B.medication C.nursing D. any specialty related to medication

18、In your opinion, what should be the least requirement of education background for clinical pharmacists?

A. Doctor’s degree B. Master’s degree C. Bachelor’s degree D. Others

19、Do you think individual medication designed by clinical pharmacists is necessary in your team?

A.very necessary B.necessary C.neutral D.basically unnecessary E.unnecessary

20、Do you think clinical pharmacists’ guidance and instruction for patients is necessary in your team?

A.very necessary B.necessary C.neutral D.basically unnecessary E.unnecessary

21、Do you think medication analysis and drug utilization evaluation conduct by clinical pharmacists are necessary in your team?

A.very necessary B.necessary C.neutral D.basically unnecessary E.unnecessary

22、Do you think medication suggestions for physicians from clinical pharmacists are necessary in your team?

A.very necessary B.necessary C.neutral D.basically unnecessary E.unnecessary

23、Do you think clinical pharmacists’ participation in medication design is necessary in your team?

A.very necessary B.necessary C.neutral D.basically unnecessary E.unnecessary

24、Do you think clinical pharmacists’ explanation of the results of blood concentration monitoring is necessary in your team?

A.very necessary B.necessary C.neutral D.basically unnecessary E.unnecessary

25、Do you think it is necessary for clinical pharmacist to monitor adverse drug reactions in your team?

A.very necessary B.necessary C.neutral D.basically unnecessary E.unnecessary

26、Do you think it is necessary for clinical pharmacist to collect and provide drug information in your team?

A.very necessary B.necessary C.neutral D.basically unnecessary E.unnecessary

27、Do you think clinical pharmacy research is necessary in your team?

A.very necessary B.necessary C.neutral D.basically unnecessary E.unnecessary

28、Do you think the staffing of clinical pharmacists in your hospital is reasonable?

A.very reasonable B.quite reasonable C.neutral

D.not reasonable E.not reasonable at all

29、Do you think clinical pharmacists are necessary in clinics?

A. Yes B.No C Not sure

If the answer is A, what is the working focus of clinical pharmacists in clinics?

（You may have less than two choices）

1. Reviewing physicians’ orders B. chronic disease management

C. medication guidance D. medication education

30、Do you think clinical pharmacists are necessary in emergency room?

A. yes B. no C. not sure

If the answer is A, what is the working focus of clinical pharmacists in clinics?

（You may have less than two choices）

A.Reviewing physicians’ orders B. chronic disease management

C. medication guidance D. medication education

31、What knowledge and skills do you think clinical pharmacists need most?

A.practical experience of clinical pharmacy

B.new knowledge and skills in pharmacy

C.knowledge and skills in clinical medicine

D.communication skills

E.academic ability of clinical pharmacy

F.others:______________

**（If there are clinical pharmacists in your working region, please answer the following question）**

32、How many hours do clinical pharmacists in your working region work every day?

A.over 6 hours/day B.4-6 hours/day C.2-4 hours/day D.less than 2 hours/day

33、What are the main duties of specialized clinical pharmacists in your working region?（You may have less than two choices）

A. drug supply and drug dispensing B. designing medical treatment

C. review physicians’ orders D. pharmacy consultation

E. pharmaceutical care F. monitoring of adverse drug reactions

G. academic research H. others

34、What is your attitude towards clinical pharmacists’ medication suggestions?

1. Totally adopt B. Partially adopt C. Neutral

D. Rarely adopt E. Absolutely do not adopt

35、Do you think clinical pharmacists are helpful to your work?

1. very helpful B. quite helpful C.not sure

D. not helpful E. be more of a hindrance than a help

36、Do you think the clinical pharmacists in your department are competent in clinical pharmacy?

A. qualified B. basically qualified C. not sure

D. only if after received training E. not qualified

**Interviewer’s name:** **Phone number:**

**Time when the form is finished:**

**Time to finish the form:**

# Questionnaire 4 A Survey on the Current Status of Pharmaceutical Care Provision in Tertiary Hospitals of China for Hospital Directors

NO:

**A Survey on the Current Status of Pharmaceutical Care Provision in Tertiary Hospitals of China**

(For Hospital Director, Administrators of Pharmacy Department)

**Part one:Basic information**

1. Name of the hospital you work for:
2. Hospital type: A. General tertiary hospital B. Specialized tertiary hospital
3. Name: Phone number:
4. Gender: A. Male B. Female
5. Age: A. 20-29 B. 30-39 C. 40-49 D. elder than 50
6. Position: A. Director B. Managing/Vice director C. Secretary
7. Deputy secretary E. Head of medical administration
8. Director of pharmaceutical department

G. Other:

1. Rank: A.Senior level B.Vice-senior level C.Intermediate level
2. Education: A. Below bachelor’s degree B. Bachelor’s degree

C. Master’s degree D. Doctor’ s degree

1. Have you ever specialized in pharmacy? A.Yes B.No

**Part two: Perception and Evaluation of the Implement of Clinical Pharmacy**

(Have only one choice unless specially notified)

1. Has your hospital been equipped with specialized clinical pharmacists?

A.Yes B.No

If the answer is B, does your hospital plan to do so?

A.Yes B.No

1. Do you think it is necessary to introduce clinical and non-clinical pharmacists to hospital after zero price difference of drugs?

A.Yes B.No

If the answer is A, then what should be the working focus of pharmacists

(Please mark a"√").

|  | Evaluation on pharmacists’ focus | | | | |
| --- | --- | --- | --- | --- | --- |
|  | Most important | Important | Neutral | Not important | Least important |
| Drug dispensing |  |  |  |  |  |
| Physicians’ order reviewing |  |  |  |  |  |
| Drug selection |  |  |  |  |  |
| Drug supply |  |  |  |  |  |
| Drug monitoring |  |  |  |  |  |
| Genetic polymorphism monitoring |  |  |  |  |  |
| Prescription evaluation |  |  |  |  |  |
| Clinical medication |  |  |  |  |  |
| Patients education |  |  |  |  |  |
| Clinical pharmacy research |  |  |  |  |  |
| Antibiotics management |  |  |  |  |  |
| Chronic diseases management |  |  |  |  |  |

1. In your opinion, what is the focus and pattern of the department of pharmacy?

(You may have less than two choices)

A.drug-centered B.patients-oriented C.focused on drug dispensing

D.focused on the rational administration of drug E.focused on drug supply F.focused on pharmaceutical services G.others:_______________

1. What do you think of the future development of hospital pharmacy?

（You may have less than two choices）

A. Mainly focus on drug supply

B. Mainly focus on drug dispensing

C. Mainly focus on academic research

D. Mainly focus on rational administration of drugs

E. Mainly focus on pharmaceutical car

1. In your opinion, what kinds of talents in pharmacy are most required for your hospital?

（You may have less than two choices）

A.dispensers B.clinical pharmacists C.pharmaceutical researcher D.pharmaceutical affairs personnel E.medicine consultant

1. Do you think clinical pharmacists are necessary in clinical environment?
2. very necessary B. necessary C.neutral

D. Unnecessary E. not necessary at all

If the answer is A or B, what are the most important duties and obligations of clinical pharmacists from your perspective of view? （You may have more than one choices）

A. drug supply and drug dispensing B. designing medical treatment

C. review physicians’ orders D. pharmacy consultation

E. pharmaceutical care F. monitoring of adverse drug effects

G. academic research H. others

If the answer is A or B, how many beds should be equipped with one clinical pharmacists?

A.10-20 B.20-50 C.50-100 D.100-200 E. more than200

1. Do you know anything about the staffing of clinical pharmacists required by NHC?

A.Yes B.No

1. According to regulations, tertiary hospitals are required to be equipped with at least 5 clinical pharmacists and secondary hospitals are required to equipped with at least 3 clinical pharmacists. In your opinion, could the staffing satisfy the needs of clinical pharmacy in hospital?

A.absolutely B.basically C.not sure D.basically not E.absolutely not

1. Do you think clinical pharmacists are necessary in clinics?

A. Yes B.No C Not sure

If the answer is A, what is the working focus of clinical pharmacists in clinics?

（You may have less than two choices）

1. Reviewing physicians’ orders B. chronic disease management

C. medication guidance D. medication education

1. Do you think clinical pharmacists are necessary in emergency room?

A. yes B. no C. not sure

If the answer is A, what is the working focus of clinical pharmacists in clinics?

（You may have less than two choices）

A.Reviewing physicians’ orders B. chronic disease management

C. medication guidance D. medication education

1. In your opinion, what education background is preferred for a clinical pharmacist?

A. pharmacy B.medication C.nursing D. any specialty related to medication

1. In your opinion, what should be the least requirement of education background for clinical pharmacists?

A. Doctor’s degree B. Master’s degree C. Bachelor’s degree D. Others

1. Do you think the clinical pharmacists in your department are competent in clinical pharmacy?

A. qualified B. basically qualified C. not sure

D. only if after received training E. not qualified

1. Do you think clinical pharmacists are helpful to your work?
2. very helpful B. a little helpful C.not sure

D. not helpful E. be more of a hindrance than a help

1. What knowledge and skills do you think clinical pharmacists need most?

A.practical experience of clinical pharmacy

B.new knowledge and skills in pharmacy

C.knowledge and skills in clinical medicine

D.communication skills

E.academic ability of clinical pharmacy

F.others:______________

1. To what degree are the following factors important for the need of clinical pharmacist? Please mark a"√".

|  | Degree of importance | | | | |
| --- | --- | --- | --- | --- | --- |
|  | Very important | Important | Neutral | A little important | Not important at all |
| Policy incentives |  |  |  |  |  |
| Hospitals’ Attention |  |  |  |  |  |
| Professional ability of the pharmacist |  |  |  |  |  |
| Acceptance of medical staff |  |  |  |  |  |
| Acknowledgement of patients |  |  |  |  |  |

1. How much do you know about NHC clinical pharmacists pilots?

A.very much B.much C.a little D.only heard of that E.never heard

1. In your opinion, in which aspect is “clinical pharmacists system” conductive?

(You may have more than one choices)

1. rational administration of drugs and improvement of public health
2. B.communication between physicians and pharmacists
3. Rational distribution of medical resources

D.realization of the patients-centered pattern E.others:________________

1. How is the "clinical pharmacist system"implemented in your hospital？

A. smooth B. basically smooth C. not sure D. not smooth E. not implemented

If the answer is E, what factors do you think handicap the implementation of clinical pharmacist system？(You may have less than two choices)

1. Policy incentives B. Environment in medical institutions

C. Attention paid by hospital leaders D. Professional ability of the pharmacist

1. Do you think it is necessary for medical institutions to apply "clinical pharmacist system"?
2. very necessary B. necessary, but the timing is not right

C. doesn’t matter D. not necessary

1. What is your attitude towards “clinical pharmacists system”?

A.strongly supportive B.supportive C.neutral

D.basically not supportive E.not supportive

1. Do you think laws and regulations are necessary to regulate clinical pharmacy work?
2. very necessary B. necessary C. neutral

D. Unnecessary E. not necessary at all

If the answer is A or B , in what aspect should the law regulate?

(You may have more than one choices)

A. make clear the role of clinical pharmacists by law

B. make clear the rights and obligations of clinical pharmacists

C. certification and employment of clinical pharmacists

D. cultivation and training of clinical pharmacists

E. assessment of clinical pharmacists

F. salaries and benefits of being a clinical pharmacists

G. others

**Interviewer’s name:** **Phone number:**

**Time when the form is finished:**

**Time to finish the form:**

# Questionnaire 5 A Survey on the Current Status of Pharmaceutical Care Provision in Tertiary Hospitals of China for Patients

NO:

**A Survey on the Current Status of Pharmaceutical Care Provision in Tertiary Hospitals of China**

(For patients)

**Part one:Basic information**

1. Gender: A. Male B. Female
2. Name: Phone number:
3. Age: A. younger than 20 B. 20-30 C. 31-40 D. elder than 50
4. Education: A. Junior high school and below
5. High school/technical secondary school
6. Junior college
7. Bachelor’s degree and above
8. Is there any clinical pharmacists in your ward area? A.yes B.no

**Part two: survey on medication habits and medicine knowledge**

1. Will you read the instruction carefully?

A.always B.sometimes C.never

1. Will you pay special attention to the contraindication, adverse drug reactions and precautions?

A.always B.sometimes C.never

1. Will you pay attention to drug’s shelf life before taking drug?

A.always B.sometimes C.never

1. If you are asked to take drugs three times every day, you will take drugs

A.after meals B.whenever I remember C.as doctor advised D.every 8 hours

1. Will you decrease the dosage when you take drugs?

A.yes B.no C.It depends

1. If adverse drug reactions occurs, you will
2. stop it and take another drug B.stop it and go to hospital

C.take it as usual D.refer to the instruction

1. If you catch a cough in life, will you take antibiotics? Eg: pennicilins(amoxicillin), cepharosporins(cefatriaxone), macrolides(erythromycin), quinolones(fluoxloxacin) ect.

A.never B.sometimes C.often D.not sure

1. If you catch a cough, will you get intravenous injections?

A.prefer intravenous injections B.only when the cough is serious

C.never, because of the danger D. follow doctor’s order

1. If the medication doesn’t work, what would you do?
2. go to doctor’s B.increase the dosage C.try another drug

D.combined medication E.continue the medication

1. Is anything required to do before taking antibiotics?
2. none B.skin test C.take other medicine first D.not sure

**Part three: perception and attitude of clinical pharmacists**

1. How much do you know about clinical pharmacy or clinical pharmacists?
2. very much B.a little C.know little but have heard

D.never heard(shift to question NO.17)

1. Where did you know clinical pharmaceutical services?

(You may have more than one choices)

A.information from hospitals at home and abroad B.hospital C.Internet D.others

1. What do you think are the main duties of clinical pharmacists?
2. drug dispensing B.prescription supervision C.adverse drug reactions monitoring D.bed checks and clinical consultation E.medication consultation
3. Would you follow the instruction provided by clinical pharmacists?

A.yes B.no

1. Do you think clinical pharmacists are important to medication?

A.very important B.important C.not important

1. Have physicians ever disagreed with clinical pharmacists in your medication?

A.yes B.no (skip question NO.22)

1. When physicians and pharmacists disagree, whose advice would you follow?

A.physicians B.pharmacists C.others

1. Who do you think should provide pharmaceutical services?
2. physicians B.pharmacists C.nursing personnel D.others

**Part four: current situation and needs of pharmaceutical care**

1. What might influence your choice of drug?

(You may have more than one choices)

1. prescription B.advertisement C.pharmacist’s recommendation

D.Your own judgement E.others

1. Where do you know about the medication knowledge?

(You may have more than one choices)

A.information on the internet B.instructions C.pharmacists D.physicians E.others

1. If the medication is not effective, what will you do?
2. consult physicians B.consultant pharmacists C.refer to the instruction

D.stop it or try another medication E.increase or decrease the dosage

1. Does your physician tell you the dosage and dosing time actively?

A.explain all the precautions B.explain some precautions C.explain nothing

1. Have you ever sought consultation from physicians about the precaution?

A.yes B.Never (skip question NO.29)

1. Are you satisfied with your physician’s explanation if you have consulted the precaution?

A.no B.fairly satisfied C.yes

1. Is the dosing time marked during drug dispensing?

A.yes B.no C.not sure

1. Will pharmacists explain the precaution for you during drug dispensing?

A.explain all the precautions B.explain some precautions C.explain nothing

1. Are you satisfied with the professional quality of pharmacists in this hospital?

A.no B.fairly satisfied C.yes D.not sure

1. Are you satisfied with the pharmacists’ attitude in this hospital?

A.no B.fairly satisfied C.yes D.not sure

1. Which one of the following choices are expected to provide pharmaceutical services?
2. face-to face consultation B.phone consultation
3. medication guidelines from hospitals D.online consultation E.others

**Part five: current situation and needs of pharmaceutical care sevices**

1. Have you ever been asked about your medical history or medication history?

A.never B.yes

(1) Do you think it is necessary? A.yes B.neutral C.no

(2) Are you satisfied with the service if any? A.no B.neutral C.yes

1. Have you ever been guided by clinical pharmacists, eg: oral dosage, dosing time and precautions?

A.never B.yes

(1)Do you think it is necessary? A.yes B.neutral C.no

(2)Are you satisfied with the service if any? A.no B.neutral C.yes

1. Have you ever sought medical consultation from clinical pharmacists?

A.never B.yes

(1)Do you think it is necessary? A.yes B.neutral C.no

(2)Are you satisfied with the service if any? A.no B.neutral C.yes

1. Have you ever been hospitalized? (If your answer is yes, then go on these questions. If your answer is no, please shift to question NO.42)

A.yes B.no

1. Has your bed been checked by clinical pharmacists during your hospitalization?

A.yes B.never

(1)Do you think it is necessary? A.yes B.neutral C.no

(2)Are you satisfied with the service if any? A.no B.neutral C.yes

1. From which aspects you expect to get medication guidance?

(You may have more than one choices)

1. instruction and dosage B.precautions C.indications and suitable crowd

D.adverse reactions and side effects E.interaction between different medicines F.first aids to relieve the unfitness caused by medication

1. Which of following choices are expected to give you the common sense of medication safety?(You may have more than one choices)

A.bulletin boards in hospital B.bulletin boards in community C.pharmacy D.physician E.clinical pharmacists F.pharmacists in pharmacy G.others:

1. Which of the following factors you think would influence the implement and quality of pharmaceutical services?(You may have more than one choices)
2. pharmacists’ professional ability B.pharmacists’ attitude

C.hospital’s attention to pharmaceutical services D.number of pharmacists E.education level of patients F.patients’ distrust for pharmacists G.others:

**Interviewer’s name:** **Phone number:**

**Time when the form is finished:**

**Time to finish the form:**

# Questionnaire 6 A Survey on the Current Status of Pharmaceutical Care Provision in Tertiary Hospitals of China for Dispensing Pharmacist

NO:

**A Survey on the Current Status of Pharmaceutical Care Provision in Tertiary Hospitals of China**

（For Non-clinical Pharmaceutical Personnel）

**Part one:Basic information**

1. Name of the hospital you work for:
2. Hospital type:A. General tertiary hospital B. Specialized tertiary hospital
3. Name: Phone number:
4. Gender: A. Male B. Female
5. Age: A. 20-29 B. 30-39 C. 40-49 D. elder than 50
6. Education: A. Below bachelor’s degree B. Bachelor’s degree

C. Master’s degree D. Doctor’ s degree

Specialty for college:­­________ Specialty for doctor’s program:­­________ Specialty for Doctors program:­­________

1. Rank:A. Junior level B. Intermediate level C. Vice-senior level D. Senior level
2. Duty:A.dispensing B.pharmaceutics C.quality control D.drug supply E.pharmaceutical research F.pharmaceutical affairs

G.pharmaceutical information H.others

1. Years of working: A.1-5 years B.6-10 years

C.11-19 years D. More than 20 years

**Part two: Perception and Evaluation of the Implement of Clinical Pharmacy**

(Have only one choice unless specially notified)

1. How much do you know about clinical pharmacy?

A.very much B.much C.a little D.have heard but know little E.never heard

1. How much do you know about the current status and future development of international clinical pharmacy?

A.very much B.much C.a little D.have heard but know little E.never heard

1. What do you think of the future development of hospital pharmacy?

（You may have less than two choices）

A. Mainly focus on drug supply

B. Mainly focus on drug dispensing

C. Mainly focus on academic research

D. Mainly focus on rational administration of drugs

E. Mainly focus on pharmaceutical care

1. Do you think clinical pharmacists are necessary in clinical environment?
2. very necessary B. necessary C.neutral

D. Unnecessary E. not necessary at all

If the answer is A or B, what are the most important duties and obligations of clinical pharmacists from your perspective of view? （You may have more than one choices）

A. drug supply and drug dispensing B. designing medical treatment

C. review physicians’ orders D. pharmacy consultation

E. pharmaceutical care F. monitoring of adverse drug effects

G. academic research H. others

If the answer is A or B, how many beds should be equipped with one clinical pharmacists?

A.10-20 B.20-50 C.50-100 D.100-200 E. more than200

1. Do you think clinical pharmacists are helpful to your work?
2. very helpful B. a little helpful C.not sure

D. not helpful E. be more of a hindrance than a help

1. Do you think the staffing of clinical pharmacists in your hospital is reasonable?

A.very reasonable B.quite reasonable C.neutral

D.not reasonable E.not reasonable at all

1. Do you think clinical pharmacists are necessary in clinics?

A. Yes B.No C Not sure

If the answer is A, what is the working focus of clinical pharmacists in clinics?

（You may have less than two choices）

1. Reviewing physicians’ orders B. chronic disease management

C. medication guidance D. medication education

1. Do you think clinical pharmacists are necessary in emergency room?

A. yes B. no C. not sure

If the answer is A, what is the working focus of clinical pharmacists in clinics?

（You may have less than two choices）

A.Reviewing physicians’ orders B. chronic disease management

C. medication guidance D. medication education

1. In your opinion, what education background is preferred for a clinical pharmacist?

A. pharmacy B.medication C.nursing D. any specialty related to medication

1. In your opinion, what should be the least requirement of education background for clinical pharmacists?

A. Doctor’s degree B. Master’s degree C. Bachelor’s degree D. Others

1. Are you optimistic about the career development of clinical pharmacists?
2. very optimistic B. optimistic C. neutral

D. not optimistic E. not optimistic at all

1. To what degree are the following factors important for the need of clinical pharmacist? Please mark a"√"

|  | Degree of importance | | | | |
| --- | --- | --- | --- | --- | --- |
|  | Very important | Important | Neutral | A little important | Not important at all |
| Policy incentives |  |  |  |  |  |
| Hospitals’ Attention |  |  |  |  |  |
| Professional ability of the pharmacist |  |  |  |  |  |
| Acceptance of medical staff |  |  |  |  |  |
| Acknowledgement of patients |  |  |  |  |  |

1. Are you interested in clinical pharmacy?

A.very interested B.interested C.neutral D.not interested E.not interested at all

1. Are you willing to become a clinical pharmacists after training?

A.strongly willing B.willing C.neutral D.unwilling E.not interested at all

1. Which one of the training for clinical pharmacists is acceptable for you?
2. full-time training B.part-time training C.correspondence education

D.online training E.others

1. How much do you know about the working patterns of clinical pharmacists?

A.very much B.much C.a little D.have heard but know little E.never heard

1. How much do you know about the duty of clinical pharmacists?

A.very much B.much C.a little D.have heard but know little E.never heard

1. What knowledge and skills do you think clinical pharmacists need most?

A.practical experience of clinical pharmacy

B.new knowledge and skills in pharmacy

C.knowledge and skills in clinical medicine

D.communication skills

E.academic ability of clinical pharmacy

F.others:______________

1. Do you think laws and regulations are necessary to regulate clinical pharmacy work?
2. very necessary B. necessary C. neutral

D. Unnecessary E. not necessary at all

If the answer is A or B , in what aspect should the law regulate?

(You may have more than one choices)

A. make clear the role of clinical pharmacists by law

B. make clear the rights and obligations of clinical pharmacists

C. certification and employment of clinical pharmacists

D. cultivation and training of clinical pharmacists

E. assessment of clinical pharmacists

F. salaries and benefits of being a clinical pharmacists

G. others

**Interviewer’s name:** **Phone number:**

**Time when the form is finished:**

**Time to finish the form:**
